# Visualizing the blind brain: brain imaging of visual field defects from early recovery to rehabilitation techniques

**DOI:** 10.3389/fnint.2014.00074

**Published:** 2014-09-30

**Authors:** Marika Urbanski, Olivier A. Coubard, Clémence Bourlon

**Affiliations:** ^1^Service de Médecine et de Réadaptation Gériatrique et Neurologique, Hôpitaux de Saint-MauriceSaint-Maurice, France; ^2^Inserm, U 1127, ICM FrontLabParis, France; ^3^CNRS, UMR 7225, ICM FrontLabParis, France; ^4^Sorbonne Universités, UPMC Univ Paris 06, UMRS 1127Paris, France; ^5^Institut du Cerveau et de la Moelle Épinière, ICM FrontLabParis, France; ^6^The Neuropsychological Laboratory, CNS-FedParis, France; ^7^Laboratoire Psychologie de la Perception, UMR 8242 CNRS-Université Paris DescartesParis, France; ^8^Service de Médecine et de Réadaptation, Clinique Les Trois SoleilsBoissise-le-Roi, France

**Keywords:** visual field defect, plasticity, cortical reorganization, rehabilitation, restoration, neuroimaging studies

## Abstract

Visual field defects (VFDs) are one of the most common consequences observed after brain injury, especially after a stroke in the posterior cerebral artery territory. Less frequently, tumors, traumatic brain injury, brain surgery or demyelination can also determine various visual disabilities, from a decrease in visual acuity to cerebral blindness. Visual field defects is a factor of bad functional prognosis as it compromises many daily life activities (e.g., obstacle avoidance, driving, and reading) and therefore the patient’s quality of life. Spontaneous recovery seems to be limited and restricted to the first 6 months, with the best chance of improvement at 1 month. The possible mechanisms at work could be partly due to cortical reorganization in the visual areas (plasticity) and/or partly to the use of intact alternative visual routes, first identified in animal studies and possibly underlying the phenomenon of blindsight. Despite processes of early recovery, which is rarely complete, and learning of compensatory strategies, the patient’s autonomy may still be compromised at more chronic stages. Therefore, various rehabilitation therapies based on neuroanatomical knowledge have been developed to improve VFDs. These use eye-movement training techniques (e.g., visual search, saccadic eye movements), reading training, visual field restitution (the Vision Restoration Therapy, VRT), or perceptual learning. In this review, we will focus on studies of human adults with acquired VFDs, which have used different imaging techniques (Positron Emission Tomography, PET; Diffusion Tensor Imaging, DTI; functional Magnetic Resonance Imaging, fMRI; Magneto Encephalography, MEG) or neurostimulation techniques (Transcranial Magnetic Stimulation, TMS; transcranial Direct Current Stimulation, tDCS) to show brain activations in the course of spontaneous recovery or after specific rehabilitation techniques.

## Introduction

Most studies interested in visual field defects (VFDs) have concentrated on the more prevalent ones. Complete homonymous hemianopia (HH) represents 70–75% of VFDs (Duquette and Baril, 2009), incomplete hemianopia (e.g., quadrantanopia) 29% of VFDs (Zhang et al., 2006a), and cerebral blindness—which is rare because it usually follows bilateral lesions—represents less than 10% of VFDs given only vascular context (Aldrich et al., 1987; Brandt et al., 2000; Niimi et al., 2008).

Principal etiologies of HH are strokes in the posterior cerebral artery territory (PCA), traumatic brain injury (TBI), and tumors (see Table 1, which displays the different etiologies reported in the literature and the percentage of associated VFDs).

**Table 1:** **Etiologies of HH and bilateral cortical blindness (bilateral CB) reported in the literature**.

		HH	Bilateral CB
PCA stroke	*N* = 904 (Zhang et al., 2006a)	69.6%	–
	*N* = 25 (Aldrich et al., 1987)	–	32%
	*N* = 332 (Brandt et al., 2000)^1^	43–74%	5–8%
TBI	*N* = 904 (Zhang et al., 2006a)	13.6%	–
	*N* = 880 (Bruce et al., 2006)	11.7%	–
Tumors	*N* = 904 (Zhang et al., 2006a)	11.3%	–
Brain surgery	*N* = 904 (Zhang et al., 2006a)	2.4%	–
Cardiac surgery	*N* = 25 (Aldrich et al., 1987)	–	20%
Demyelination	*N* = 904 (Zhang et al., 2006a)	1.4%	–
Cerebral angiography	*N* = 25 (Aldrich et al., 1987)	–	12%

*N, number of patients included in the studies; VFDs are given in percentages in each study; –, not reported in the study*.

After a stroke, 45% of the lesions involved the occipital lobes and 32.2% the optic radiations (Zhang et al., 2006a); after TBI 12.5% of lesions involved the occipital lobes and 23.2% in association with a lesion of the optic radiations (Bruce et al., 2006). Most VFDs occurred after a lesion in the primary visual cortex (V1) although a lesion in the early extrastriate areas has been exceptionally reported to give rise to VFDs (e.g., patient with a lesion in ventral V3 and V4 presenting a right upper homonymous quadrantanopia, Slotnick and Moo, 2003; patients with a lesion in MT+/V5 presenting motion blindness or “akinetopsia”, Zeki, 1991; Zihl et al., 1991; Vaina et al., 2001).

In France, there have been very few studies on the recovery of VFDs, or if they exist, they rely on relatively small samples. In the UK, a recent review reported visual loss in 45–67% of patients in the acute phase of the stroke, and in the long term for 8–25% of patients following adjustment for recovery of visual field (Rowe et al., 2013). Concerning HH, about 90,000 to 120,000 new cases per year both in the US and in Europe are reported by Sahraie (2007). Ajina and Kennard (2012) reported 30% of patients at the acute phase of the stroke (Haerer, 1973) and 8–26% of patients left with persistent HH (Gray et al., 1989; Gilhotra et al., 2002). About 6% of patients are left with cerebral blindness at a chronic stage (Zihl, 2000).

In this review, we will focus on studies in human adults with acquired VFDs, in which a neuroimaging technique (Positron Emission Tomography, PET; Diffusion Tensor Imaging, DTI; functional Magnetic Resonance Imaging, fMRI; MagnetoEncephalography, MEG) or a neurostimulation technique (Transcranial Magnetic Stimulation, TMS; transcranial Direct Current Stimulation, tDCS) has been employed to document brain changes. We will first present neuroimaging studies documenting the changes in the brain in the context of spontaneous recovery and then other studies documenting the changes in the brain after the use of a rehabilitation technique. For the purpose of this review, we only included VFDs in the context of postgeniculate lesions. Eye diseases or optic nerve pathologies were excluded. Studies in lesioned animals, in children or employing only perimetries or questionnaires to document the improvement of VFDs were also not included in this review.

## Early spontaneous recovery

Full spontaneous recovery of VFDs is rare: 5% (Duquette and Baril, 2009); 10% patients with hemianopia within the first 2 weeks (Gray et al., 1989; Pambakian and Kennard, 1997). However, many studies have shown that a partial and early spontaneous recovery may occur after brain injury although its quickness is still being debated.

Zhang et al. (2006b) have shown that in 254 patients with hemianopia followed over a 15-year period, 50–60% chances of recovery occurred at 1 month, 20% at 6 months, whereas no patient improved after 6 months. Similarly, Perez et al. (2009) reported that in 101 patients with hemianopia, 40% had spontaneous recovery within 3 months though the recovery only concerned a part of the visual field, consistent with other prospective studies showing improvement only in the peripheral zones of the lower quadrants (Celebisoy et al., 2011) or limited to 3–7 degrees of visual angle depending on the extent of sparing in the affected hemifield (Zihl, 2000).

Recovery seems to be limited to the first 6 months after the injury and may also depend on the lesion site. Bosley et al. (1987) showed that the impairment of glucose metabolism in the striate cortex measured with PET did not change over time in three patients with HH after a lesion involving V1 whereas it improved over time in two patients with HH after lesion involving extrastriate areas sparing V1. This improvement in striate metabolism was associated with a recovery of their VFDs, contrary to the former three patients. Thus recovery is restrictive in time and in space (Zhang et al., 2006b) because the spontaneous plasticity following V1 damage may be related to changes in the properties of neural circuits beyond the lesion and to a decreased inflammation around the lesion site during the first few weeks after damage (Huxlin, 2008).

Neuroimaging studies have attempted to document the neural changes during spontaneous early recovery. Raposo et al. (2011) studied eight patients with an infarct in the posterior cerebral artery territory presenting VFDs, who were examined within the first month of the stroke, 1 month later and 3 months later. Five of eight patients had restricted V1 ventral lesion and three of eight had V1 ventral and dorsal damage. Patients underwent a neurological, neuropsychological and ophthalmological examination, visual behavioral study (conscious color and motion perception) and fMRI (visual stimuli targeting motion and color presented separately within each hemifield). The authors reported that color and motion vision recovery were complete or subcomplete 1 month after the onset of the stroke. At the acute phase, there was no ipsilesional V1 activation for color or motion stimuli, while it appeared in color perception at follow-up. There was also nonspecific bilateral V4 activation in color task and nonspecific contralesional MT+/V5 activation in motion task. With time, activations in MT+/V5 and V4 bilaterally became more specific and correlated with performance.

Polonara et al. (2011) reported the case of a 24-year-old woman with left hemianopia who underwent fMRI and DTI in the acute phase and 1 month after an ischemic stroke involving the right calcarine cortex. At the acute phase, ipsilesional V1 did not show any activation when peripheral stimulation was presented in the left hemifield. The mean fractional anisotropy (FA) measured by DTI in the ipsilesional optic radiations was reduced compared with the left hemisphere. At 1-month follow-up, both right and left V1 elicited comparable activations in response to stimulation in the contralateral hemifield. Mean FA in the optic radiations was more similar in both hemispheres. Similarly, Yoshida et al. (2006) reported the case of a 68-year-old man presenting right hemianopia after an infarction of the left extrastriate areas who underwent fMRI and DTI over the ensuing 12 months. Functional magnetic resonance imaging was acquired 2 days, 9 days, 30 days and 1 year after the onset of the stroke. The results showed that larger areas of left cortical activation were activated progressively and that the asymmetry between the activations of both hemispheres decreased. Diffusion tensor imaging was acquired 2 days, 9 days and 1 year after the onset of the stroke and a tractography of the optic radiations was performed. At 2 days after onset, fiber tracking was completely interrupted in the left side due to the cortical lesion, whereas 1 year later left optic radiations could be reconstructed by fiber tracking. The authors concluded that the initial larger recruitment of cortical areas in the intact hemisphere decreased with recovery along with a progressive increasing activation in the lesioned hemisphere.

In summary, spontaneous recovery of VFDs occurs during the first 6 months following brain damage (mostly due to stroke and TBI) with a peak in recovery after 1 month. Consistently, functional brain imaging using PET, fMRI or DTI shows progressive activation of cortical and subcortical areas with increasing recovery, which correlates with psychophysical performance. The lesion side may have differential effects on recovery and prognosis.

## Residual vision in the chronic phase

At a more chronic phase after brain injury (usually after 6 months after the lesion), VFDs become more stable and one can observe some phenomena of residual vision in the affected visual field. Studies in monkeys and later in humans have demonstrated visual function persistence even when the primary visual cortex had been destroyed (Cowey and Stoerig, 1995; Weiskrantz, 2004; Stoerig, 2006). Contrary to anosognosia where patients are unaware of their deficits (e.g., Anton’s syndrome, for review see Bisiach and Geminiani, 1991), patients with blindsight are aware of their deficits but unaware of their intact functions. For example, patients could perform visual discrimination in the blind hemifield though they persisted in saying that they could not see anything. This phenomenon, called “blindsight”, has been first described in patient DB in whom right primary visual cortex was removed and the left visual field (LVF) was defected (Weiskrantz, 1986, 1996). According to Weiskrantz, blindsight may be defined as “a visual discrimination in the absence of awareness” and may be separated in two sub-types: (1) Type I for an unconscious version: patient seemed to be able to detect visual target aspects without any conscious awareness of the stimulus presented and (2) Type II for a residual vision accompanied by a level of awareness: patients reported a feeling that something happened or moved without real visual experience.

According to the nature of the task performed, a different classification of blindsight capacities was proposed (Danckert and Rossetti, 2005). Action-blindsight was used to refer to patients who were able to localize a stimulus not consciously perceived in blind field by pointing or making saccades; attention-blindsight was used to refer to patients who were able to discriminate the direction of motion of a stimulus without action and who have the feeling that something happened in the visual field. Finally, ‘Agnosopia’ defined abilities to discriminate forms or colors without conscious awareness (Zeki and Ffytche, 1998).

Evidence for basic residual visual motion (Barbur et al., 1980; Weiskrantz, 1986; Schoenfeld et al., 2002; Morland et al., 2004), shape (Barbur et al., 1993; Stoerig and Cowey, 1997; Goebel et al., 2001), and color in the blind field (Stoerig, 1987; Barbur et al., 1998; for a review see Huxlin, 2008) has been reported in the literature consistently.

More recently, evidence for residual vision has been demonstrated for non basic visual properties of stimuli such as category discrimination (patient DB in Trevethan et al., 2007; patient TN with bilateral CB following two consecutive occipital strokes less than 2 months interval in Van den Stock et al., 2013) or navigation skills (patient TN in de Gelder et al., 2008).

Another type of blindsight has been described and has led to many interesting studies: the affective blindsight, in which patients are able to process emotional cues presenting in their blind hemifield (de Gelder et al., 1999). Pegna et al. (2005) studied patient TN using fMRI and showed that he was able to guess the valence of the facial expressions (positive/negative) on photographs presented to him. This ability correlated with the activity of his right amygdala, consistent with the results of Morris et al. (2001) and of Tamietto and de Gelder (2008) in patient GY, who is a right hemianopic patient extensively studied. Affective blindsight has been shown in studies using fMRI for the perception of dynamic whole-body emotional expressions (patient GY in Van den Stock et al., 2011), for perception of body and facial emotional expressions (patient TN in Van den Stock et al., 2014) and for perception of gaze direction (patient TN in Burra et al., 2013).

However blindsight is not present in all patients with VFDs and depends on particular neurophysiological properties of subcortical structures (the superior colliculus and the pulvinar which receive visual information from the magnocellular pathway). Indeed, blindsight is sensitive to the temporal characteristics of the stimuli (5–20 Hz in Sahraie et al., 1997; 10–33 Hz inTrevethan and Sahraie, 2003), to the spatial channel of the stimuli (low spatial frequency <3.5 cycles/degree in Sahraie et al., 1997, 2010; Trevethan and Sahraie, 2003), facilitated by an increase in stimulus size (Sahraie et al., 1997) or for high contrast stimuli (Ffytche et al., 1995), but insensitive to short wavelength (Tamietto et al., 2010). Therefore, different underlying anatomical mechanisms have been proposed to explain blindsight and many studies have concentrated on the brain activations associated with this phenomenon.

### Spared cortical islands of V1

Some authors have postulated a direct relation between the preserved portion of striate areas and blindsight in the corresponding visual field (Fendrich et al., 2001; Morland et al., 2004).

Specifically Morland et al. (2004) used fMRI with static, moving and flickering stimuli in seven hemianopes and one patient with a clearly spared region of the visual field in an otherwise blind hemifield. Their findings support the existence of small, spared active regions of V1 that mediate residual vision in some patients, consistent with the results of Raposo et al. (2011). However, some patients with V1 damage showed no activation in the striate cortex in fMRI but could still present a blindsight phenomenon, which does not support the hypothesis of the existence of spared islands in V1 to account for blindsight (Kentridge et al., 1997; Sahraie et al., 1997; Stoerig et al., 1998; Zeki and Ffytche, 1998; Ptito et al., 1999; Goebel et al., 2001; Morland et al., 2004). The existence of spared islands of V1 has been considered to be responsible for conscious visual perception (Celesia et al., 1991; Fendrich et al., 1992; Stoerig and Cowey, 1995) but many studies have later demonstrated that visual awareness could be present in the absence of a healthy V1 (Barbur et al., 1993; Ffytche et al., 1996; Zeki and Ffytche, 1998; Morland et al., 1999; Kleiser et al., 2001; Ffytche and Zeki, 2011).

### Extra-geniculostriate pathways (or subcortical routes)

Another finding in Morland et al. (2004) is the existence of residual motion direction discrimination in patients in whom a lesion ruled out the hypothesis of spared cortical islands of V1. For these patients, the existence of other pathways bypassing V1 has been implicated to account for residual motion direction consistent with results of Celesia et al. (1991) and Ptito et al. (1999).

**Figure 1:**
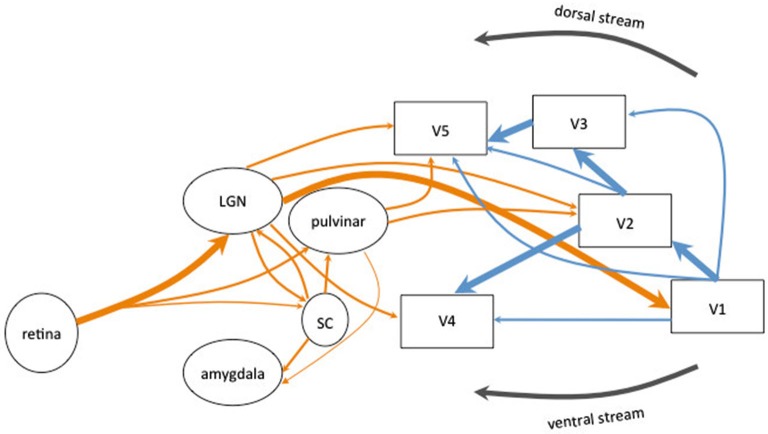
**Schematic diagram of the human visual system**. The main connections originating in the retina are represented in thick arrows. They synapse in the lateral geniculate nucleus (LGN) and project to the primary visual cortex (V1). V1 sent information to the extrastriate areas (V2, V3, V4 and MT+/V5). Most of the corticocortical (in blue) and subcortico-cortical (in orange) connections are reciprocal but are not represented for clarity of the schema. Alternative pathways are represented in thin arrows. The extrageniculostriate pathway belonging to the dorsal visual stream, originates in the retina and synapses in the superior colliculus (SC) and in the pulvinar and projects directly to extrastriate areas (in particular area MT+/V5) bypassing both V1 and the LGN. This pathway has been accounted to mediate action blindsight. Another colliculo-pulvinar pathway, associated with the ventral visual stream, synapses in the LGN and projects to extrastriate areas (in particular area V4) bypassing V1. This pathway has been accounted to mediate color and shape residual discrimination. Other collicular pathways are represented: the colliculo-pulvinar pathway (between SC and pulvinar), the pulvino-amygdalar pathway (between the pulvinar and amygdala) and the colliculo-pulvino-amygdalar pathway (between the SC, the pulvinar and the amygdala). These pathways have been accounted to mediate affective blindsight.

The existence of two sub-cortical pathways, one associated with the dorsal visual stream and the other with the ventral visual stream (Goodale and Milner, 1992), bypassing V1 and reaching directly the extrastriate cortex have been implicated in different blindsight classifications (see Figure 1). Retinal projections to the superior colliculi (SC) and the pulvinar bypass both V1 and dorsal lateral geniculate nucleus (LGN) to project to area MT+/V5, which is part of the dorsal visual stream (Bittar et al., 1999; Ptito et al., 1999; Schoenfeld et al., 2002). This colliculo-pulvinar pathway has been involved in accounting for residual motion discrimination and in the patients’ ability to make accurate saccadic eye movements to localize stimuli. Superior colliculi allows to drive visually-guided behavior without awareness and would be involved in the reflexive orienting of attention (Rafal et al., 1988), the unconscious visual processing and attentional orienting (Kentridge et al., 1997). Action-blindsight may be underlied by the projections from the pulvinar to MT+/V5 whereas attention-blindsight may be underlied by the projections from the colliculo-pulvinar pathway to the posterior parietal cortex. However, both types seem to be closely related and may rely on the same neural pathways associated with the dorsal stream (Danckert and Rossetti, 2005).

Since collicular neurons do not have color opponency (Stoerig and Cowey, 1989, 1991; Ro and Rafal, 2006), a second pathway projecting directly from the LGN to V4 and MT+/V5 (especially demonstrated in monkeys in Sincich et al., 2004; for a review see Huxlin, 2008) has been implied to account for the residual color discrimination (Stoerig, 1987; Barbur et al., 1998; Bridge et al., 2010) and form discrimination in patients (Barbur et al., 1993; Stoerig and Cowey, 1997; Goebel et al., 2001). This route projecting to V4 and associated with the ventral visual stream has been referred to account for agnosopia (Zeki and Ffytche, 1998).

Another subcortical pathway has been implied to account for affective blindsight. Morris et al. (2001) have shown that the activation of the amygdala of patient GY for unseen faces correlated with activity in the SC and the pulvinar, suggesting the existence of a colliculo-pulvino-amygdalar pathway, whose existence has been demonstrated by Tamietto et al. (2012) using DTI tractography.

### Interhemispheric connections (callosal and non callosal)

Residual vision can not only be mediated by sub-cortical pathways but also by the reorganization in the ipsilesional and/or the contralesional hemisphere (Baseler et al., 1999; Bittar et al., 1999; Goebel et al., 2001; Bridge et al., 2008, 2010), allowing for the processing of visual information either ipsilaterally or bilaterally.

**Figure 2:**
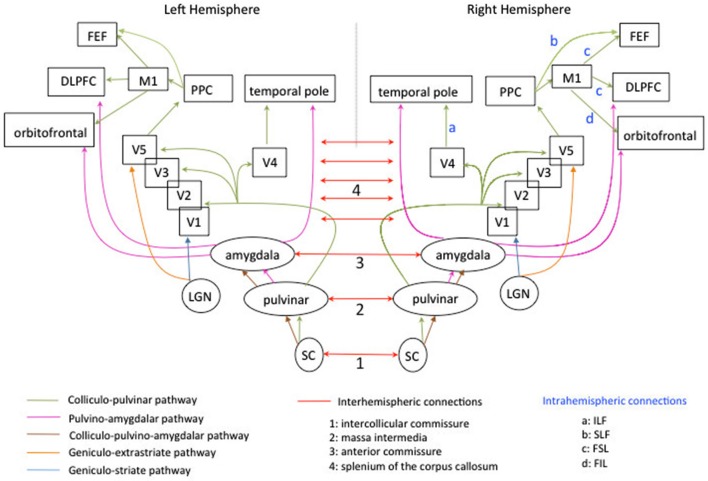
**Schematic diagram of the subcortico-cortical connections, subcortical connections intra-and interhemispheric connections mediating blindsight in the human left and right hemispheres**. For the clarity of the display, not all subcortical connections are represented. The colliculo-pulvinar pathway is represented in green. It extends to several subcortical and cortical areas and is highly symmetrical in the two hemispheres. Fibers passing through the superior colliculus (SC) and pulvinar continue to V1 and extrastriate areas, temporal pole, posterior parietal cortex (PPC), primary motor cortex (M1), frontal eye-field (FEF), dorsolateral prefrontal cortex (DLPFC) and orbitofrontal cortex. A part of the bundle passes through the amygdala, the caudate and brainstem (not represented). The pulvino-amygdalar pathway is represented in pink and is also symmetrical in the two hemispheres. Fibers passing through the pulvinar and the amygdala extend to temporal pole, dorsal prefrontal cortex, orbitofrontal cortex. It also extends to caudate and SC (not represented). The colliculo-pulvino-amygdalar pathway is represented in maroon and is symmetrical in the two hemispheres. It does not extend to other cortical or subcortical areas (see Tamietto et al., 2012).The geniculo-extrastriate pathway is represented in orange and is symmetrical in the two hemispheres. Only fibers connecting the LGN to area MT+/V5 are represented for clarity of the schema. The geniculostriate pathway is represented in blue and is symmetrical in the two hemispheres. Only fibers connecting the LGN to area V1 are represented. The complexity of cortico-cortical and subcortico-cortical routes is partially represented in Figure 1. The interhemispheric connections are represented in red. Both SC are connected via the intercollicular commissure (n°1), both pulvinar via the massa intermedia (n°2; see Catani and Thiebaut De Schotten, 2012), both amygdala are connected by the anterior commissure (n°3). Cortical areas in the occipital cortex are connected by the splenium of the corpus callosum (n°4). Intra-hemispheric connections are labeled by letters in light blue. They are displayed only for the right hemisphere but are also present in the left hemisphere. The pathway linking V4 to temporal pole is the inferior longitudinal fasciculus (ILF: a). The pathway linking the PPC to FEF is the superior longitudinal fascisculus (SLF: b). The pathway linking M1 to FEF and the DLPFC is the superior frontal longitudinal fasciculus (SFL: c). The pathway linking M1 to the orbitofrontal cortex is the inferior frontal longitudinal fasciculus (FIL: d).

Neuroimaging studies using DTI have suggested that this redistribution of cerebral activations may rely on interhemispheric connections (Silvanto et al., 2007; Bridge et al., 2008). Specifically, Bridge et al. (2008) studied patient GY, five healthy controls and one age-matched male who underwent diffusion weighted-MRI and fMRI. GY exhibited similar to the controls bilateral tracts between LGN and MT+/V5. However, in GY, the ipsilateral pathways between LGN to V1 were bilaterally smaller than the pathway between LGN to extrastriate areas (see Figure 2, in blue and orange). The authors found two differences in GY’s brain compared to controls: the presence of prominent bilateral tracts from the splenium to MT+/V5 (suggesting an increased cortico-cortical connectivity between these extrastriate areas through callosal connections) and the presence of a contralateral pathway between right LGN and left extrastriate areas.

These differences in connectivity pattern in GY have also been documented by Tamietto et al. (2012) using DTI tractography to reconstruct three pathways previously identified in healthy controls (see Figure 2). The colliculo-pulvinar pathway in GY’s damaged hemisphere (in green Figure 2) was reduced in strength and failed to extend to frontal areas, especially to area 46 which has been implicated in conscious perception (Sahraie et al., 1997), consistent with the absence of conscious emotion perception in GY (Tamietto and de Gelder, 2010). Moreover, the connections from contralateral posterior areas in GY’s intact hemisphere were strengthened, consistent with an increase of interhemispheric connections after V1 damage. Connections of the pulvino-amygdalar pathway in the damaged GY’s hemisphere (in pink Figure 2) also extended more posteriorly to visual areas than to frontal regions compared to controls. The connections of the colliculo-pulvino-amygdalar pathway were strengthened in the damaged hemisphere of GY (in maroon Figure 2), consistent with the role of this pathway in affective blindsight, for which information did not depend directly from V1.

Bridge et al. (2010) studied patient SBR with bilateral damage to the gray matter of V1 sparing the adjacent white matter and surrounding visual areas. Using a “motion” task in fMRI contrasting moving dots with stationary dots, they found a bilateral activation of areas MT+/V5 despite no significant activation of V1. The tracts between LGN and V1 appear to show some degeneration while tracts between LGN and V5 did not differ from controls. The authors had previously reported similar findings in patient GY (Bridge et al., 2008) but in patient SBR, the very specific lesion suggested that ipsilateral connection between LGN and MT+/V5 may be particularly important for residual function.

Using fMRI, Perez et al. (2013) presented images of natural scenes filtered (in high and in low frequencies or non filtered) to right and left hemianopes who where asked to perform a detection and a categorization tasks. They showed a different pattern of reorganization depending on the lesion side. The right hemianopes (left occipital lesion) seemed to have a predominant intra-hemispheric reorganization whereas the left hemianopes (right occipital lesion) a predominant inter-hemispheric reorganization, suggesting that hemispheric specialization (visuospatial abilities for the right hemisphere and language abilities for the left hemisphere) could be present at this early level.

Neurostimulation studies using TMS have also highlighted the importance of interhemispheric connections in VFDs and their recovery. Silvanto et al. (2007) used TMS and reported that GY experienced visual sensation of phosphenes in his blind field only when bilateral stimulation were applied over MT+/V5. According to the authors, this conscious sensation can only be conveyed by the contribution from GY’s intact hemisphere to explain why stimulation of the damaged hemisphere can reach awareness. These findings also suggested the presence of an increased connectivity via transcallosal connections, consistent with the tractography results obtained by Bridge et al. (2008). In a subsequent study in TMS performed on GY, Silvanto et al. (2009) showed that TMS applied over the area MT+/V5 in the damaged hemisphere modulated the appearance of phosphenes induced from V1 in the intact hemisphere (contrary to control subjects whose TMS over area MT+/V5 never influence the phosphenes induced from V1 in the other hemisphere). This finding was consistent with the abnormal functional connectivity between GY’s both hemispheres. However in their previous study (Silvanto et al., 2007), GY experienced bilateral phosphenes, consistent with a role of interhemispheric connections between extrastriate areas in both hemispheres and with the increased anatomical connectivity documented in Bridge et al. (2008). In this study (Silvanto et al., 2009), GY never perceived bilateral phosphenes because the combined stimulation of the left V5/MT+ and the right V1 did not induce phosphene in his blind field. Actually, GY had atrophy of callosal fibers in the forceps major which is part of the splenium (Silvanto et al., 2007), but it has been argued that non callosal pathway can mediate this interhemispheric transfer, although more slowly (Ffytche et al., 2000).

The existence of interhemispheric non callosal connections such as the intercollicular commissure and the ipsilateral and contralateral projections from the SC to various brain areas may explain why patients with bilateral occipital lesions (involving the splenium of the corpus callosum) or with a unilateral lesion impairing the splenium may nonetheless present blindsight (see Figure 2).

To summarize, residual vision is present after the first 6 months following brain damage. Blindsight refers to the ability of VFDs’ patients to perform well in tasks involving eye movements, pointing, reaching, prehension, discrimination, identification, emotional processing, though they have no consciousness of their performance. Functional brain imaging shows that the retino-collicular pathway is more likely to account for blindsight than cortical islands in V1. Interhemispheric connections (either callosal between homologous visual areas, either non callosal connections via subcortical structures) may also play an important role in blindsight phenomena and variability, which is compatible with the differential effect of the lesion side on residual vision due to distinct underlying plasticity mechanisms.

## Cortical reorganization/Plasticity

Some studies have demonstrated that the retinotopic organization of V1 could be preserved even if the visual cortex is damaged (Baseler et al., 1999; Ho et al., 2009; Reitsma et al., 2013). However, in some cases, patients can also present an atypical organization of their visual cortex after brain injury (Reitsma et al., 2013). In 27 patients with clear anatomical evidence of damage involving visual cortex and/or underlying white matter, Reitsma et al. (2013) presented three patients with an expanded ipsilateral field representation compared with healthy controls, whereas 22/27 patients had a typical retinotopic organization. For the authors, this atypical organization could rely on the unmasking of the interhemispheric suppression from the intact visual cortex due to the lesion that would in turn unmask retino-geniculate afferents representing the vertical meridian and ipsilateral visual field. They acknowledged other plausible mechanisms such as axonal sprouting and synaptogenesis in the deafferented visual cortex in association with the strengthening of long-range excitatory connections.

Some neuroimaging studies have also reported cases to document reorganization and plasticity in the visual system. Plasticity could be viewed as a recruitment of the contralesional hemisphere (and therefore interhemispheric connections), which has been demonstrated in the early stage of recovery (Raposo et al., 2011) and in more chronic stages (Nelles et al., 2002) where it could also mediate blindsight (Zeki and Ffytche, 1998; Ffytche et al., 2000; Silvanto et al., 2007; Bridge et al., 2008).

Plasticity could also be viewed as a recruitment of activity by nearby healthy cortex (Baseler et al., 1999), a disinhibition of pre-existing long-range horizontal connections within V1, sprouting of new horizontal connections in V1, which have been demonstrated in animals studies (Darian-Smith and Gilbert, 1994, 1995; Das and Gilbert, 1995) or changes in the functional interactions between higher-level visual cortical areas and V1 (Huxlin, 2008). Processes of plasticity may have different time courses, which overlap, from synaptic gain in the short term to axonal sprouting and new circuits properties in the long term (Wandell and Smirnakis, 2009).

Goebel et al. (2001) used fMRI and retinotopic mapping in two patients with long-standing left VFDs history (FS and GY) to compare the responsiveness of dorsal and ventral stream areas after stimulation of both hemifields. They found that GY’s ipsilesional extrastriate areas responded to stimulation to either hemifield. The authors proposed that these findings were consistent with a kind of plastic changes of the system compensating for the loss of V1 (which is normally the major source of MT+/V5 input) in GY.

Baseler et al. (1999) performed an fMRI study in GY. They found that the foveal stimulation in the lesioned occipital lobe exhibited normal retinotopic organization as GY’s lesion spared the foveal representation. The stimulation of the blind VF exhibited a different topography of GY’s extrastriate areas depending on the stimulus configuration (full or annular wedge), which now responded to positions restricted near the lower vertical meridian. Their findings suggested the involvement of subcortical projections to extrastriate cortex, transcallosal projections (consistent with the restricted activity around the lower vertical meridian) and residual inputs from V1 near the margin of the lesion. They assumed that because V2 neurons in GY’s lesioned occipital lobe were deprived of their V1 input, they were colonized by other neurons in neighboring cortex. The colonization could be mediated by “strengthening or disinhibition of long-range connections or by the creation of new connections” (Das and Gilbert, 1995) through plastic reorganization.

Ioannides et al. (2012) studied patient GY and three healthy controls with MEG using a distributed source model to estimate the spatiotemporal properties of neural activity following the presentation of checkerboard pattern stimuli in different portion of the visual field. In control subjects, activity started in the first 100 ms in V1 and spread through dorsal and ventral streams in the next 100 ms towards extrastriate areas. In GY’s damaged hemisphere no activity was detected before 130 ms. The first activity detected was in the ipsilesional extrastriate cortex (around the middle occipital gyrus, the middle temporal gyrus and the superior temporal sulcus) and spread towards higher level areas and backward to early retinotopic visual areas. Moreover, the back-propagated activity did not follow the retinotopic organization and did not have well-defined response peaks. Again, these findings in GY may be due to plastic reorganization following long-term lesion.

Dilks et al. (2007) reported the case of a patient with a stroke sparing V1 but affecting the right inferior optic radiations (which normally provide information to V1 from the upper field). The patient was blinded in the upper quadrant of the LVF but he also exhibited a distorted perception of the intact lower visual field (stimuli appeared vertically elongated). Six months after the onset of the stroke, the patient underwent behavioral testing and retinotopic mapping-fMRI, which revealed that this perceptual distortion was mirrored by a distorted visual field map in V1. They found that the regions normally dedicated to the representation of the upper LVF were now activated by lower LVF stimuli due to the upper quadrantanopia. Thus the regions of V1 representing the lower quadrant of the LVF were expanded, leading to an expanded representation of the left horizontal meridian and to the perceptual distortion. Huxlin (2008) proposed that the perceptual plasticity exhibited by the patient reported by Dilks et al. (2007) could include “dis-inhibition of pre-existing long-range horizontal connections within V1, sprouting of new connections in V1 or changes in the functional interactions between higher-level visual cortical areas and V1” (Huxlin, 2008).

Schoenfeld et al. (2002) have studied color changes and motion direction discrimination to target integrity of the ventral and the dorsal visual streams in a patient suffering from a left hemorrhagic PCA stroke, who underwent both fMRI and MEG recordings. They found activation following motion and color-change stimuli in the hemianopic field in several extrastriate areas of the lesioned hemisphere. The MEG recordings provided evidence for activation first in V5 in the lesioned hemisphere with other extrastriate areas being activated later. In the intact hemisphere, V1/V2/V3 activity preceded V4/V5/V8 activity whereas in the lesioned hemisphere, motion and color stimuli activated first V4/V5/V8 regions. The authors proposed that the cortical reorganization after a V1 lesion may involve a change of connectivity between extrastriate areas (V4/V8 and V5), and a change in the dominant direction of flow of visual information between areas spared by the lesion, with V5 playing a key role in distributing subcortical signals to other extrastriate regions via feedback and feedforward connections already in place (Hupe et al., 1998). Indeed, the existence of recurrent loops between higher and early visual areas has been demonstrated in animals and in human studies (Hupe et al., 1998; Goebel et al., 2001; Schoenfeld et al., 2002; Silvanto et al., 2005). These loops have been shown to amplify and focus activity of neurons in lower-order areas (Hupe et al., 1998) and are supposed to organize neuronal activity into stable resonant states and could be a neural correlate of conscious vision (e.g., Tononi and Edelman, 1998; Engel and Singer, 2001; Goebel et al., 2001; Silvanto et al., 2005).

However there are controversies about plasticity and cortical reorganization *per se*. Indeed, in most of these studies reported previously (except that of Ho et al., 2009, in which the patient was studied 1 year post stroke; that of Dilks et al., 2007, in which the patient suffered from a stroke 6 months before the study; and that of Reitsma et al., 2013, in which 2/3 patients with abnormal retinotopic organization had acquired VFDs during adulthood), all the patients had a long-standing history of VFDs, because the injury had occurred earlier in their lives, thus probably allowing for a better reorganization (Goebel et al., 2001; Haak et al., 2014). Alternative explanations have been proposed to account for cortical reorganization.

Rather than cortical reorganization or remapping, the findings may be more accurately accounted for by properties of neuronal receptive fields and modulatory feedback signals from extrastriate areas (Haak et al., 2012, 2014). For example, according to Haak et al. (2014) the term “reorganization” implies the presence of long term anatomical changes (Wandell and Smirnakis, 2009). Thus some cases of abnormal activity, such as that observed by Dilks et al. (2007), could be explained on the basis of intrinsic neuronal properties that surface only when the normal input signal is absent. When neurons are deprived from their original input, the feedback signals from the far periphery of the VF become visible as a distorsion of the visual field map and thus affect perception (Haak et al., 2014). Papanikolaou et al. (2014) used fMRI to measure the population receptive field (pRF) properties in area V1 in five patients with partial or complete quadrantanopia after a lesion in V1 or in optic radiations. They showed that in two patients, some pRF centers shifted their location near the border of the scotoma. Moreover, the pRF size in the spared V1 cortex of patients was increased in both the damaged and the healthy hemispheres, suggesting the recruitment of area nearby the lesion and a reorganization of the subcortical inputs from subcortical structures (LGN, pulvinar).

Different hypotheses have been advanced to account for cortical/subcortical reorganization and underlying plasticity mechanisms: nearby cortex recruitment, disinhibition of long-range connections, sprouting of new connections, interactions between V1 and higher level visual areas, or modulatory feedback signals. Far from being exclusive, we suggest that these different mechanisms are likely to co-occur and their effects to interact resulting in complex and variable solutions within the damaged brain. Visual field defects are associated with bad functional prognosis because they impair daily activities (driving, reading, social activities, leisure or work, fall risk, accidental risk and quality of life) (Trauzettel-Klosinski, 2011). To counteract VFDs’ deleterious impact, different rehabilitation programs have been proposed.

## Reorganization after rehabilitation

Plasticity in the central nervous system after brain injury may play a key role in restitution and may be enhanced through training of visual functions (Pöppel et al., 1978; Zihl and von Cramon, 1985). Based on experiments in primates with VFD after brain injury (Cowey, 1967; Mohler and Wurtz, 1977), visual training aims at restoring or at compensating visual blind field. Though some authors have postulated that lost visual functions cannot be recovered (Horton, 2005b), others have emphasized the critical role of rehabilitation techniques such as visual retraining or field stimulation in the recovery of VFDs (Popelreuter, 1917; Preobrazenskaya cited in Luria, 1963; Zihl and von Cramon, 1982). Three main approaches may be considered for rehabilitation. The first employs compensatory techniques by using intact visual abilities to improve natural adaptation strategies (i.e., eye movements training, see Bolognini et al., 2005; Roth et al., 2009; Schuett et al., 2009). The second concerns optical aids, using relocation of the visual field with monocular or binocular prisms (Peli, 2000; Bowers et al., 2008; Ross et al., 2012). Finally restorative therapy (reported in this review) has been proposed, using training stimulation programs to increase the blind visual field directly.

### Rehabilitation by borderzone stimulation

The first promising studies of training (Zihl and von Cramon, 1979, 1985; Zihl, 1981) proposed external stimulation between normal and impaired visual field (partially defective area called transition zone or borderzone) in patients with damage in the geniculostriatal visual system. Results highlighted an increase in seeing visual field size in patients with cerebral blindness. Kasten and Sabel (1995) proposed a standardized and automatic program called “Visual Restoration Therapy” (VRT, NovaVision^©^). Rehabilitation consisted of a home-based program to be performed on a computer (Kasten and Sabel, 1995). The region between the intact and damaged visual fields along the vertical meridian (borderzone) was also targeted by the therapy. Patient with VFD had to maintain a central fixation on the screen device and respond by pressing a key whenever a light stimulus appeared. These training exercises were performed twice daily for half an hour for 6 months.

However VRT remained criticized because of the variability of results reported in different studies. In most cases reported, visual expansion did not exceed 5% (Kasten and Sabel, 1995; Kasten et al., 1998b) and was less pronounced in others studies (from 1° to 6.7°) (Pommerenke and Markowitsch, 1989; Kerkhoff et al., 1992, 1994) or even absent (Balliet et al., 1985). Some authors suggested that visual field improvements could be an artefact of eye movements (Horton, 2005a; Reinhard et al., 2005) and that controversial results could be related to (1) the perimeter techniques employed; (2) lesion localization; or (3) explanatory mechanisms (Pouget et al., 2012).

Behavioral VRT studies have described a visual field expansion after this rehabilitation and proposed an explanatory mechanism based on the reactivation of residual neuronal activity in the ischemic transition zone through the expansion of the receptive field of small spared neural structures (Kasten et al., 1998a). The borderzone has been characterized as an area of suboptimal visual perception corresponding to surviving neurons (Kasten et al., 1998a; Sabel et al., 2011). Thus Pleger et al. (2003) have shown an increase of BOLD signal in perilesional primary visual cortex after 6 months of rehabilitation in three patients with cortical blindness. Julkunen et al. (2003) explored rehabilitation at three times (before and after visual field training and after a follow up of 3 months) one patient with right homonymous upper quadrantanopia after left occipito-temporal lesion. Perimetry, subjective evaluation and visual evoked potential to right hemi-field stimulation improved during the training period and were maintained at the follow-up (specifically with a 5–10° increase in visual field). Positive correlation between changes in rCBF and changes in perimetry results was found in the contralesional occipital area. Similarly, a cohort of six chronic right hemianopic patients with a left temporal or occipital lesion underwent fMRI before and 1 month after beginning VRT (Marshall et al., 2008). Results reported a modification of brain activity correlated with a relative improvement in response time for detection of the stimuli in the borderzone after therapy, in secondary and associated visual areas, right dorsolateral prefrontal cortex, bilateral anterior cingulate cortex, and bilateral basal ganglia. Positive correlation was also observed and the authors concluded that VRT could induce a modification of brain activity associated with a process of shift in spatial attention (from the seeing location toward the borderzone location). More recently, Raemaekers et al. (2011) have explored the properties of visual cortex (V1, V2, and V3) before and after VRT by using fMRI and perimetry in eight chronic patients with visual field defect. Vision restoration therapy induced visual field recovery as measured with perimetry (mean increase of 3.94°) and fMRI results showed a shift of receptive fields to a higher eccentricity and some growth of receptive field size. However, no evidence for extensive representation of regained visual field was found.

Moreover, based on motor recovery after training rehabilitation combined with tDCS, (Plow et al., 2011, 2012) proposed a visual rehabilitation (VRT) associated with tDCS. They tested two patients in chronic phase with left occipital lesion and right VFD (Plow et al., 2011). One patient received VRT combined with active tDCS and the other received VRT combined with sham tDCS (no stimulation). The anode electrode was placed with the intention to stimulate the occipital cortex bilaterally. Objective perimetry pre- and post-treatment showed a greater expansion in the visual field border after rehabilitation in patients with VRT combined with active tDCS (expansion of 3.55° in the central visual field and a 4° shift inward from the periphery). These results suggested that the stimulation of the occipital cortex with tDCS during VRT promoted visual rehabilitation. Functional magnetic resonance imaging data were also obtained in this patient following rehabilitation and revealed activation in perilesional and in bilateral higher area (V2/V3 and MT+/V5), consistent with reactivation surviving visual area hypothesis. However, it seems difficult at this time to disentangle the contributory effect of VRT from tDCS. Plow et al. (2012) confirmed these previous results by proposing the same protocol to 12 patients and suggested that tDCS associated with VRT accelerate the recovery in accuracy detection (Halko et al., 2011; Plow et al., 2012). The exact underlying mechanism of tDCS in humans is not known. It is supposed that tDCS may enhance VRT effects by modulating excitability of surviving visual networks including perilesional area but also bilateral higher visual areas (for a review see Valero-Cabré et al., 2011).

### Rehabilitation by blindsight

The ability to perform visual discrimination in the absence of awareness (Weiskrantz et al., 1974) opened up new horizons for neuro-visual rehabilitation (Ro and Rafal, 2006). As described in Section Residual vision in the chronic phase, blindsight may suggest the existence of a residual visual treatment process after striate pathway injury whereby visual information may travel through LGN directly to extrastriate cortical areas (Sincich et al., 2004; Bowers et al., 2008; Bridge et al., 2010). Thus Vanni et al. (2001) trained one patient (MR) with a right posterior medial cerebral infarct and left hemianopia to detect flickering luminance patterns (disk and letter). Magneto encephalography results showed a right attenuated transient occipital response and a prominent response in the right superior temporal cortex. The authors concluded that the input of the superior temporal cortex might come through the SC and pulvinar and compensate for the impaired input of the primary visual cortex. Consistent with these results, Raninen et al. (2007) reported the case of two patients (KS and IT) with a left occipital lesion, who were trained with a detection task of a flickering light stimulus and a letters identification task. Neuromagnetic responses (at 1–2 months intervals) of patient KS showed the strongest response in the ipsilesional posterior superior temporal area. In contrast, the strongest response of the other patient (IT) was in the controlesional occipital area. Henriksson et al. (2007) showed with an fMRI mapping that both visual hemifields were represented in IT’s intact hemisphere and more specifically in the MT+/V5 area, in a region around the superior temporal sulcus and in visual areas V1, V2, V3 and V3a. Implication of interhemispheric connections appeared required. However, the minor activation observed in the left hemisphere excluded a pathway through callosal connections as if a disconnection of the left occipital regions from visual processing occurred. Inter-commissural connection of the SC, connecting left hemisphere activity to the right extrastriate visual areas via the pulvinar seemed to be a more plausible explanation.

To summarize, depending on whether theoretical and physiological frameworks have pointed out the role of either cortical or subcortical pathways in residual vision, rehabilitation techniques in VFDs have privileged either cortical borderzone stimulation or blindsight training. In both cases, significant improvements in visual performance have been observed. However, the underlying mechanisms are not well understood, which could be disentangled by more systematic functional brain imaging studies testing more patients.

## Conclusion

Visual field defects following post-geniculate lesion, among which 70–75% are HH and 29% quadrantanopia, are common neurological disorders after stroke or traumatic brain injury, with high deleterious impact on activities of daily life: walking, driving, reading, etc. Spontaneous recovery only occurs within the 6 months following brain damage, and most of the recovery is done after 1 month. After 6 months, almost no spontaneous recovery may occur and rehabilitation techniques have been developed to improve residual vision. Depending on theoretical frameworks and physiological considerations underlying visual capabilities, strategies have alternatively given importance to either borderzone stimulation (the stimulation through behavioral training or transcranial stimulation of cortical areas in the neighborhood of damaged primary visual cortex) or blindsight (the stimulation of spared subcortical routes). Visual improvements have been documented in both cases. However, from a phenomenological point of view, there is a great variability regarding the success of the rehabilitation according to the patients. Some patients seem to “see” in their blind field and feel more confident in their perception (Chokron et al., 2008), leading to a better quality of life. Subcortical processes, which have been involved in mediating blindsight, have been shown to be non conscious but they might interact with cortical processes via an integration of their activity with activity in cortical structures, which in turn exert a feedback to subcortical structures (Tamietto and de Gelder, 2010). Nonetheless, subcortical processes seem to be distinct from cortical processes in terms of sensory threshold, time-scale and in terms of main connections to cortical areas implicated in conscious perception.

Most of rehabilitation techniques are specific to the type of stimuli used in training and therefore non-transferable to other stimuli present in real life. Moreover, they require repetitive training over an extended period (Ajina and Kennard, 2012). As example, Rowe et al. (2013) have shown that in 479 patients with visual loss treated with different options (visual search training, visual awareness, typoscopes, substitutive prisms, low vision aids, refraction, and occlusive patches), only 7.5% had full recovery, 39% had improvement and 52% did not recover at follow-up.

Underlying physiological mechanisms of spontaneous recovery and/or rehabilitation interventions as well as their interactions are not well understood in humans. In this context, functional brain imaging examining gray and white matter in brain-damaged patients is the only objective tool to understand further plasticity and compensatory mechanisms of visual loss, recovery and rehabilitation. Learning plays a crucial role in plasticity because practice has a role in facilitating recovery and reorganization (Levin, 2006). For example, studies using perceptual learning in control subjects have demonstrated changes in receptive field properties within early visual cortex and increases in activation (Bao et al., 2010; Frank et al., 2014). Brain imaging evidence of perceptual learning has shown cortical and white matter changes, which took place quickly and efficiently. Gray matter changes may rely on dendritic spine growth and synapse turnover (Barnes and Finnerty, 2010; May, 2011) whereas white matter changes may be based on larger-scaled axonal remodelling and increased myelination (Johansen-Berg et al., 2012; Ditye et al., 2013; Lövdén et al., 2013). As cortical reorganization or plasticity might rely on this type of changes in the functional architecture and RF properties in visual areas (Gilbert and Li, 2012), promising studies should perhaps use perceptual learning in patients (see Huxlin et al., 2009, who have trained seven patients with HH to discriminate complex motion direction in their intact and blind field). The success or the failure may depend on the pattern of cortical damage and the involvement of damage to subcortical and interhemispheric connections (Reitsma et al., 2013).

## Conflict of interest statement

The authors declare that the research was conducted in the absence of any commercial or financial relationships that could be construed as a potential conflict of interest.

## Footnotes

^1^This article review reported results from 4 series of PCA stroke leading to a total of 322 patients. Percentage ranges correspond to the minimal and maximal percentages of VFDs reported by Brandt et al. (2000).
